# What Is Required for Neuronal Calcium Waves? A Numerical Parameter Study

**DOI:** 10.1186/s13408-018-0064-x

**Published:** 2018-07-13

**Authors:** Markus Breit, Gillian Queisser

**Affiliations:** 1 G-CSC, Goethe University Frankfurt, Frankfurt am Main, Germany; 2 Department of Mathematics, Temple University, Philadelphia, USA

**Keywords:** Calcium waves, Endoplasmic reticulum, Ryanodine receptors, 3D modeling, Structure-function interplay, Numerical simulation

## Abstract

Neuronal calcium signals propagating by simple diffusion and reaction with mobile and stationary buffers are limited to cellular microdomains. The distance intracellular calcium signals can travel may be significantly increased by means of calcium-induced calcium release from internal calcium stores, notably the endoplasmic reticulum. The organelle, which can be thought of as a cell-within-a-cell, is able to sequester large amounts of cytosolic calcium ions via SERCA pumps and selectively release them into the cytosol through ryanodine receptor channels leading to the formation of calcium waves. In this study, we set out to investigate the basic properties of such dendritic calcium waves and how they depend on the three parameters dendrite radius, ER radius and ryanodine receptor density in the endoplasmic membrane. We demonstrate that there are stable and abortive regimes for calcium waves, depending on the above morphological and physiological parameters. In stable regimes, calcium waves can travel across long dendritic distances, similar to electrical action potentials. We further observe that abortive regimes exist, which could be relevant for spike-timing dependent plasticity, as travel distances and wave velocities vary with changing intracellular architecture. For some of these regimes, analytic functions could be derived that fit the simulation data. In parameter spaces, that are non-trivially influenced by the three-dimensional calcium concentration profile, we were not able to derive such a functional description, demonstrating the mathematical requirement to model and simulate biochemical signaling in three-dimensional space.

**Electronic Supplementary Material:**

The online version of this article (10.1186/s13408-018-0064-x) contains supplementary material.

## Introduction

Intracellular calcium signals define a transition point between electrical signals and biochemical responses in neurons. While basal calcium concentrations in the cytosol are very low, neurons can modulate local cytosolic calcium concentrations to induce microdomain calcium signals, which can integrate to produce longer ranging signal propagation towards the soma. There they reach the nucleus and trigger gene transcription responses relevant for learning [1–4] and neuroprotection [5–8]. Cellular calcium signals are shaped by calcium transport mechanisms embedded in the plasma membrane, across which calcium can be bi-directionally exchanged between cytosol and the extracellular space (see [9] for an overview). In addition, intracellular organelles like mitochondria and the endoplasmic reticulum (ER) function as large calcium stores [10–16]. Organelle membranes are equipped with calcium exchange mechanisms that allow transport of calcium from the cytosol into the organelles or vice versa. Some of these mechanisms—notably the ryanodine receptor channel (RyR) in the ER membrane, which is able to release large amounts of calcium from the ER into the cytosol—have a positive feedback property: Their opening is facilitated by the presence of cytosolic calcium and will trigger the release of even more calcium through surrounding channels. This calcium-induced calcium release mechanism (CICR) overcomes the limited reach of purely diffusive calcium signals in a buffered regime. CICR in neurons has been implicated in various neurodegenerative diseases [17–23] and has been studied with respect to CICR wave-like properties [24–38]. The importance of CICR wave-like dynamics therefore merits thorough investigation. One major question is how the cellular and intracellular architecture can shape and regulate calcium signals that are able to propagate over long distances, for instance in the context of synapse-to-nucleus communication. It was shown in [26] that variations in the activation of Ca^2+^ store release through inositol 1,4,5-trisphosphate (IP_3_) receptors were able to significantly change the calcium wave patterns. Additionally, the distances between distinct IP_3_ receptor clusters and the pump strength were shown to control wave stability and instability [37, 38]. Sequestration properties through SERCA pumps [36] and mitochondrially controlled CICR [27] were further implicated in CICR. Related work has shown that calcium signals can modulate the shape of the cell nucleus [39, 40]. Thus, there seem to exist mechanisms in which calcium signals shape the geometry of organelles and the intracellular architecture shapes calcium signals.

**Fig. 1:**
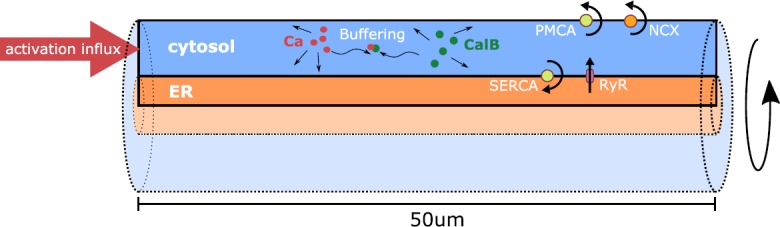
Simulation domain and model components. The domain for all simulations of this study was a cylindrical dendrite 50 μm in length with variable radius containing a centrally positioned cylindrical ER of variable radius. The rotational symmetry of the domain was used to reduce the problem to two dimensions (axial and radial position). The calcium model contains calcium in the cytosol and the ER as well as calbindin (CalB) in the cytosol. The dynamics of both are governed by a diffusive process and a buffering reaction. Calcium can cross the ER membrane through RyR channels and SERCA pumps, the plasma membrane through PMCA and NCX pumps. Each simulation was initiated by a 1 ms calcium influx through the left end of the cytosol

In this study, we therefore chose to investigate how the relationship between ER and dendrite geometry can influence the dynamics of CICR waves. The ER is typically thought of as a continuous extension of the outer nuclear envelope that forms the rough ER surrounding the cell nucleus. As smooth ER, it extends into dendrites, all the way into synaptic spines [41]. Reconstructions of portions of smooth ER in dendrites have shown the ER to define a moderately branched, continuous structure [41, 42]. From an idealized standpoint, the ER has previously been considered as a neuron-within-a-neuron [43–45], approximated in form of a cable that is embedded in a larger cable (the dendrite). We decided to follow this path of morphology simplification and designed perfectly symmetrical ER/dendrite cable-in-cable model domains (see Fig. 1).

The associated calcium model consists of a diffusion-reaction partial differential equation for the propagation of calcium in the cytosol and the reaction of free calcium with the buffer calbindin. Endoplasmic calcium is modeled by a simple diffusion equation. Membrane exchange mechanisms are included via flux boundary conditions at the plasma membrane and the ER membrane: Ca^2+^-ATPase pumps, Na^+^/Ca^2+^ exchangers, as well as a leakage term on the plasma membrane; sarco-/endoplasmic reticulum Ca^2+^-ATPase pumps, ryanodine receptor channels and a leakage flux on the ER membrane.

Our study focused on: (1) how calcium dynamics depend on the parameters dendrite radius, ER radius, and ryanodine receptor density, (2) whether thresholds can be found for stable calcium waves and (3) on gathering information about wave velocities. Simulation results show how cellular architecture, even in this simplified scenario, can determine the shape and fate of calcium waves and their stability. These data were further used to derive empirical laws that explain the behavior of the observed calcium dynamics in distinct geometric regimes. As a side note and in agreement with [26], we also show that the higher spatial dimension (as compared to classical 1D cable models) is indeed required to adequately represent even the most basic properties of calcium wave propagation.

## Model and Methods

### Model Domain

We conducted all simulations on a perfectly cylindrical model dendrite with a fixed length of 50 μm and variable radius, containing a cylindrically shaped ER of variable radius positioned exactly at the center of the dendrite; see Fig. 1. We used the rotational symmetry to reduce the problem to two dimensions. Calcium signals were induced by a calcium influx density through the left boundary of the dendrite with an initial strength of $2.5\times 10^{-18} \ \mathrm{mol}\ \upmu \mathrm{m}^{-2}\ \mathrm{s}^{-1}$ that linearly decreased to zero within 1 ms.

### Model Equations

Spatio-temporal calcium dynamics in the intracellular space are modeled by a system of diffusion-reaction equations described in the following. Transmembrane currents through channels and pumps are incorporated into these equations as flux boundary conditions (cf. Sect. 2.3).

Our model includes cytosolic/endoplasmic calcium and, in the cytosol, calbindin-D_28k_ (CalB) as a calcium buffer. In the equations, we will represent cytosolic and endoplasmic calcium concentrations by the symbols $c_{c}$, $c_{e}$, and the (unbound) buffer concentration by *b*, respectively. Mobility in the cytosol/ER is described by the diffusion equation

1 $$\begin{aligned} \frac{\partial u}{\partial t} = \nabla \cdot ( D \nabla u ), \end{aligned}$$

where $u(x,t)$ represents both calcium and calbindin as functions in space and time.

The buffering reaction between cytosolic calcium and calbindin is described by

2 $$\begin{aligned} \mathrm{Ca}^{2+}+\mathrm{CalB} \mathop{\rightleftharpoons} _{\kappa_{b}^{-}}^{\kappa_{b}^{+}} \bigl[\mathrm{CalBCa}^{2+}\bigr]. \end{aligned}$$

The fact that CalB has four distinct high-affinity calcium-binding sites [46] is taken into account by quadrupling its concentration for the simulation (thus implicitly assuming identical and independent binding sites). The full domain equations for cytosolic calcium and calbindin are thus given by

3 $$\begin{aligned} \frac{\partial c_{c}}{\partial t} & = \nabla \cdot ( D_{c} \nabla c_{c} ) + \bigl( \kappa_{b}^{-} \bigl( b^{\mathrm{tot}}-b \bigr) - \kappa_{b}^{+} b c_{c} \bigr) , \end{aligned}$$

4 $$\begin{aligned} \frac{\partial b}{\partial t} & = \nabla \cdot ( D_{b} \nabla b ) + \bigl( \kappa_{b}^{-} \bigl( b^{\mathrm{tot}}-b \bigr) - \kappa_{b}^{+} b c_{c} \bigr) \end{aligned}$$

in the cytosolic domain, where the concentration of the CalB-Ca^2+^ compound is expressed by the difference of the total concentration of CalB present in the cytosol ($b^{ \mathrm{tot}}$) and free CalB, the former of which is assumed to be constant in space and time (this amounts to the assumption that free and calcium-binding CalB have the same diffusive properties). All diffusion and reaction parameters are listed in Table 1.

**Table 1:** Model parameters and initial values

*Initial and equilibrium values*		
$c_{c}$	50 nM	(chosen)
$c_{e}$	250 μM	(chosen)
$c_{o}$	1 mM	(chosen)
$b^{\mathrm{tot}}$	40 μM	[47]
*Diffusion/reaction*		
$D_{c}$	220 $\upmu \mathrm{m}^{2}\ \mathrm{s}^{-1}$	[48]
$D_{b}$	20 $\upmu \mathrm{m}^{2}\ \mathrm{s}^{-1}$	[49]
$\kappa _{b}^{-}$	19 $\mathrm{s}^{-1}$	[47]
$\kappa _{b}^{+}$	27 $\upmu \mathrm{M}^{-1}\ \mathrm{s}^{-1}$	[47]
*RyR channel*		
$k_{a}^{-}$	28.8 $\mathrm{s}^{-1}$	[50]
$k_{a}^{+}$	1500 $\upmu \mathrm{M}^{-4}\ \mathrm{s}^{-1}$	[50]
$k_{b}^{-}$	385.9 $\mathrm{s}^{-1}$	[50]
$k_{b}^{+}$	1500 $\upmu \mathrm{M}^{-3}\ \mathrm{s}^{-1}$	[50]
$k_{c}^{-}$	0.1 $\mathrm{s}^{-1}$	[50]
$k_{c}^{+}$	1.75 $\mathrm{s}^{-1}$	[50]
$I_{R}^{\mathrm{ref}}$	$3.5 \times 10^{-18}\ \mathrm{mol}\ \mathrm{s}^{-1}$	[51] (approx.)
*SERCA pumps*		
$I_{S}$	$6.5 \times 10^{-21}\ \mathrm{mol}\ \upmu \mathrm{M}\ \mathrm{s}^{-1}$	[52], (adapt.)
$K_{S}$	180 nM	[24]
*PMCA pumps*		
$I_{P}$	$1.7\times 10^{-23}\ \mathrm{mol}\ \mathrm{s}^{-1}$	[53]
$K_{P}$	60 nM	[54]
$\rho _{P}$	500 $\upmu \mathrm{m}^{-2}$	(estim.)
*NCX pumps*		
$I_{N}$	$2.5 \times 10^{-21}\ \mathrm{mol}\ \mathrm{s}^{-1}$	[53], (adapt.)
$K_{N}$	1.8 μM	[53]
$\rho _{N}$	15 $\upmu \mathrm{m}^{-2}$	(estim.)
*Leakage*		
$v_{l,e}$	38 nm $\mathrm{s}^{-1}$	(calc.)
$v_{l,p}$	4.5 nm $\mathrm{s}^{-1}$	(calc.)

### Membrane Transport Mechanisms

In order to study calcium waves, we included ryanodine receptors for calcium release from the ER. We also added sarco-/endoplasmic reticulum Ca^2+^-ATPase pumps (SERCA) for re-uptake as well as a leakage flux on the ER membrane. Moreover, we defined fluxes across the plasma membrane by addition of Ca^2+^-ATPase pumps (PMCA), Na^+^/Ca^2+^ exchangers (NCX) and a plasma membrane leakage term. This amounts to the ER and plasma membrane flux density equations

5 $$\begin{aligned} j_{\mathrm{ERM}} &= j_{R} - j_{S} + j_{l,e}, \end{aligned}$$

6 $$\begin{aligned} j_{\mathrm{PM}} &= - j_{P} - j_{N} + j_{l,p}. \end{aligned}$$

Here, $j_{R}$ is the RyR flux density, $j_{S}$ the SERCA flux density and $j_{l,e}$ the leakage flux density on the ER membrane. The flux densities of PMCA, NCX and the leakage flux density of the plasma membrane are denoted by $j_{P}$, $j_{N}$ and $j_{l,p}$, respectively. All flux densities are oriented towards the cytosol so that positive signs mean influx into the cytosol. Besides RyR, ER-membrane-bound IP_3_ receptor (IP_3_R) channels are another source for intracellular calcium. They are known to require calcium and, more importantly, IP_3_ in the cytosol to become permeable to luminal calcium. IP_3_ is produced at the plasma membrane by receptor activation of phospholipase C. Compared to RyR calcium efflux, IP_3_R calcium dynamics take place on a slower time scale (governed by the diffusion of IP_3_). Preliminary simulations for the present study have shown that IP_3_Rs may contribute to the initiation of a traveling wave, when small calcium influxes are not enough to trigger a wave through RyRs alone. The wave dynamics are, however, dominated by RyR. We therefore decided to exclude IP_3_R-mediated calcium dynamics.

To incorporate existing single-channel or single-pump models in the partial differential equations formulation, we assume channels and pumps to be continuously distributed along the membranes, using their membrane densities to calculate the flux densities $j_{\mathrm{ERM}}$ and $j_{\mathrm{PM}}$.

#### RyR Channels

The calcium flux density through ryanodine receptor channels in the ER membrane is given by an expression of the form

7 $$\begin{aligned} j_{R} = \rho_{R} \cdot p^{o}_{R} \cdot I_{R}, \end{aligned}$$

where $\rho_{R}$ is the density of RyR in the ER membrane, $p^{o}_{R}$ is the open state probability of a single channel, and $I_{R}$ the single-channel Ca^2+^ current. We describe the single channel ionic current by

8 $$\begin{aligned} I_{R} = I_{R}^{\mathrm{ref}} \frac{c_{e} -c_{c}}{c_{e}^{ \mathrm{ref}}}, \end{aligned}$$

where the reference current $I_{R}^{\mathrm{ref}}$ is approximated from data presented in [51].

The open probability for RyR channels is modeled according to [50] and can be calculated as the sum of the two open states $o_{1}$ and $o_{2}$ in the system of ordinary differential equations

9 $$\begin{aligned} o_{1} &= 1 - c_{1} - o_{2} - c_{2}, \end{aligned}$$

10 $$\begin{aligned} \frac{\partial c_{1}}{\partial t} &= k_{a}^{-} o_{1} - k_{a} ^{+} c_{c}^{4} c_{1}, \end{aligned}$$

11 $$\begin{aligned} \frac{\partial o_{2}}{\partial t} &= k_{b}^{+} c_{c}^{3} o_{1} - k_{b}^{-} o_{2}, \end{aligned}$$

12 $$\begin{aligned} \frac{\partial c_{2}}{\partial t} &= k_{c}^{+} o_{1} - k_{c} ^{-} c_{2} \end{aligned}$$

emerging from a four-state Markov model with the kinetic constants $k_{a}^{\pm }$, $k_{b}^{\pm }$ and $k_{c}^{\pm }$.

#### SERCA Pumps

The current from sarco-/endoplasmic reticulum Ca^2+^-ATPase pumps is described by a model from [24], which was adapted for the three-dimensional case, and gives rise to the Ca^2+^ flux density

13 $$\begin{gathered} j_{S} = \rho_{\mathrm{S}} \cdot \frac{I_{S}c_{c}}{ ( K_{S}+c _{c} ) c_{e}}. \end{gathered}$$

The model reflects the dependence of the Ca^2+^ current not only on the cytosolic concentration, but also on the endoplasmic saturation. The density $\rho_{\mathrm{S}}$ of SERCA pumps in the ER membrane was adapted to the RyR channel density in each simulation to ensure a zero net flux through the membrane in equilibrium conditions.

An interesting question with regard to the modeling of trans-membrane currents is their equilibration at stationary, i.e., resting concentrations. The usual approach is to calibrate a leakage current such that it exactly counters the combined net current of all other involved mechanisms at equilibrium. At the ER membrane, this is only possible as long as the resulting direction of the leakage current points outward. With varying RyR densities, however, this is not necessarily the case (as leakage and RyR current have the same direction). Since the SERCA current is the only inward current through the ER membrane that we consider in our model, we chose to calibrate their density to ensure a zero net flux at equilibrium. This results in variable SERCA densities (larger when the RyR density is larger), which appears plausible from an energy consumption point of view: An increase in leakage currents to counter a reduction in RyR density would only lead to an increase in “futile cycling” through leakage and SERCA pumps. Another approach to avoid unneccessary consumption of energy is to use a bi-directional SERCA model (cf. [55], also for a more detailed view on “futile cycling”). Our simulations showed that the exact equilibration of the membrane currents has only a minuscule impact on the results presented here—the threshold values presented in the Results section only change by about 1% with equilibration by leakage flux and constant SERCA density (data not shown). The reason is that the propagation of the wave front takes place on a much faster time scale than the re-uptake of calcium through SERCA pumps. The same applies to the extrusion by PMCA and NCX pumps, which are therefore modeled in a basic way.

#### NCX and PMCA Pumps

For the Na^+^/Ca^2+^ exchanger current, our model assumes a constant Na^+^ concentration at the plasma membrane. The current densities for both plasma membrane transport mechanisms are expressed as first- and second-order Hill equations, respectively (cf. [53]):

14 $$\begin{aligned} j_{P} &= \rho_{P} \cdot \frac{I_{P} {c_{c}}^{2}}{K_{P}^{2}+{c_{c}} ^{2}}, \end{aligned}$$

15 $$\begin{aligned} j_{N} &= \rho_{N} \cdot \frac{I_{N} c_{c}}{K_{N}+c_{c}}. \end{aligned}$$

#### Leakage

Both ER and plasma membranes allow a leakage flux not accounted for by the above transport mechanisms. The plasma membrane leakage flux is calibrated to ensure zero membrane net flux in the equilibrium state. Leakage flux densities are modeled by

16 $$\begin{aligned} j_{l,e} &= v_{l,e} \cdot ( c_{e}- c_{c} ) , \end{aligned}$$

17 $$\begin{aligned} j_{l,p} &= v_{l,p} \cdot ( c_{o} - c_{c} ) , \end{aligned}$$

where $c_{o}$ is the extracellular Ca^2+^ concentration, which is assumed to be constant throughout all simulations. Values for all model parameters are gathered in Table 1.

### Numerical Methods

For numerical simulations, the three equations are discretized in space using a finite volumes method. This allows for a natural integration of current densities across ER and plasma membranes into the reaction-diffusion process.

The system of ordinary differential equations (in time) arising from this procedure is nonlinear (due to the nonlinear reaction term and, more importantly, the highly nonlinear transport terms across the membranes). As these equations, notably the RyR channel dynamics, require a precise calibration of the time step size used for numerical solution, we employed a linearly-implicit extrapolation (LIMEX) scheme to solve the nonlinear system, since LIMEX offers automated error-estimate-based control of integration order and step size [56, 57].

For the results we present here, the linear problems emerging in the LIMEX method were solved using a Bi-CGSTAB [58] linear solver preconditioned by a geometric multigrid method using Gauss–Seidel smoothing and SuperLU [59] as base solver. Computations were facilitated by a domain decomposition parallelization approach and carried out using the UG 4 framework [60] on the JURECA computer system at the Jülich Supercomputing Centre [61].

### Implementation

All model components were implemented in a NeuroBox project. NeuroBox [62] is a neuroscientific toolbox that combines 1D, 2D and 3D modeling and simulation of electrical and biochemical signaling in a visual workflow environment. Visual workflows are created with VRL-Studio [63] and the general-purpose numerical framework UG 4 [60] is used to solve the set of coupled nonlinear partial differential equations.

## Results

### Range of Calcium Waves

In a first series of simulations, we examined how calcium dynamics depend on the ER radius and the density of RyR channels in the ER membrane. To that end, we created five model dendrite domains with a fixed dendrite radius of 0.2 μm each and an ER radius of 40 nm, 50 nm, 60 nm, 70 nm, and 80 nm, respectively. We then simulated calcium dynamics with RyR densities ranging from 0.5 $\upmu \mathrm{m} ^{-2}$ to 4.0 $\upmu \mathrm{m}^{-2}$ on all five domains and measured how far the initial calcium signal was able to travel through the dendrite.

**Fig. 2:**
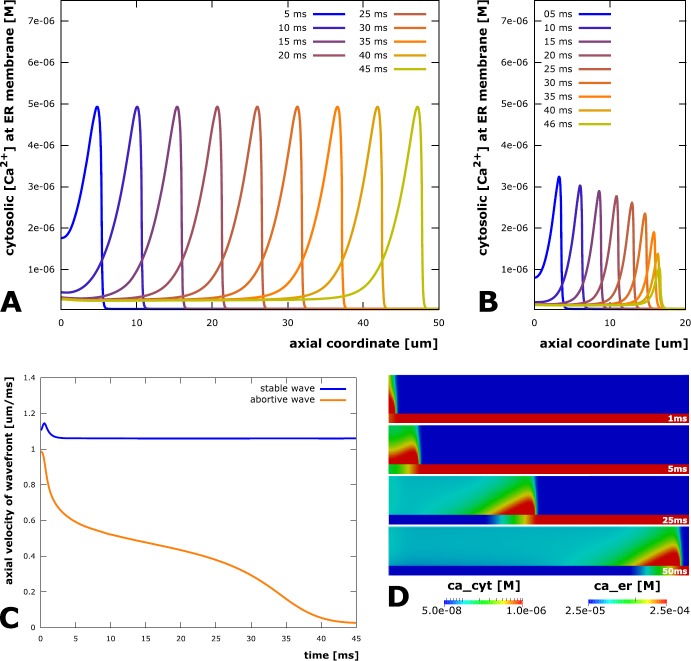
Propagation of stable and abortive calcium waves. (**A**) Axial calcium profiles of a stable calcium wave at different points in time (dendrite radius 0.4 μm, ER radius 0.15 μm, RyR density 2.5 $\upmu \mathrm{m}^{-2}$). The depicted cytosolic calcium profiles are recorded directly at the ER membrane and over the whole length of the model dendrite. The shape of the wave remains constant and travels from left to right in a convective manner, although driven by a reaction-diffusion process with nonlinear calcium exchange across the ER. (**B**) Axial calcium wave profiles of an abortive calcium wave (same setup as in A, but with a smaller ER radius of 0.11 μm). While a wavefront traveling from left to right is clearly visible, it has a smaller amplitude than in A and breaks down before reaching the far end of the dendrite. (**C**) Velocity of the calcium wave fronts in A and B as a function of time. The stable wave quickly reaches a constant velocity of about 1.06 $\upmu \mathrm{m}\ \mathrm{ms}^{-1}$ and travels through the whole dendrite at this speed, while the velocity of the abortive wave peaks at about 0.98 $\upmu \mathrm{m}\ \mathrm{ms}^{-1}$ and then declines to zero while the wave breaks down. (**D**) Sample simulation of a 1 μm dendrite with elicitation of a stable calcium wave. The radial coordinate is scaled by a factor of 8 to enhance visibility

The propagation distance was defined as the distance between the left end of the dendrite (the activation point) and the rightmost position where the open probability of the RyR channels exceeded 0.1 at any point in time. In our study we observe both stable and abortive calcium waves. The qualitative differences are illustrated in Fig. 2. For a stable wave (depicted for dendrite radius 0.4 μm, ER radius 0.15 μm, RyR density 2.5 $\upmu \mathrm{m}^{-2}$ in Fig. 2A), the traveling wavefront is clearly visible and maintains a constant shape. Reduction of the ER radius to a value of 0.11 μm makes the wave regime abortive (Fig. 2B): The wave front does not propagate further than 20 μm and the concentration at the wave front diminishes over time. While the velocity of the abortive wave decays to zero, it remains constant in the stable case (Fig. 2C)—justifying the assumption that the stable wave can propagate arbitrarily far. Snapshots of a sample simulation of a stable wave are depicted in Fig. 2D).

**Fig. 3:**
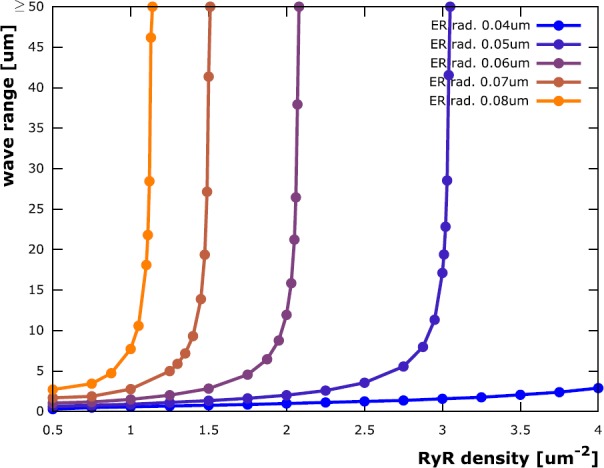
Range of calcium waves. Simulations on a cylindrical model dendrite (radius 0.2 μm) containing a cylindrical ER (radius 40 nm, 50 nm, 60 nm, 70 nm, 80 nm) reveal that there is a threshold RyR density (at the singularities of the plotted curves) above which calcium waves are stable. Decreasing the RyR density below the threshold induces abortive waves, whose travel distances rapidly decline with RyR density reduction. Larger ER domains lead to a lowering of the stabilizing RyR density threshold (singularity moves to the left), indicating that the ability to sustain a calcium wave depends on the rate at which calcium can be released from the ER

We plotted the propagation distance as a function of RyR density in Fig. 3. The traces for ER radii of 50 nm, 60 nm, 70 nm and 80 nm rise slowly at first, but then very rapidly approach a singularity at a RyR density that is specific to each domain. This threshold RyR density decreases with increasing ER radius and also exists for an ER radius of 40 nm, but is outside the plotted RyR density range in Fig. 3.

We draw the following conclusions from these observations for our 0.2 μm model dendrite:

(i) A minimal RyR density is required in all five domains to elicit a calcium wave. The sharp gradient in the curves depicted in Fig. 3 show that once a critical RyR density is surpassed, calcium waves become stable. Otherwise the initial calcium influx causes only a very localized transient that does not propagate along the dendrite. This result is comparable to minimal distance criteria for the IP_3_ receptor clusters studied in [37].
(ii) Dendrites with smaller ER need a higher RyR density to elicit a wave, indicating that the ability to trigger a calcium wave scales with the rate of calcium release from the ER. This seems to agree with [37], where wave stability is controlled by IP_3_R pump strength and, as mentioned above, by IP_3_R cluster distances. The critical threshold for wave stability decreases with increasing ER radius. Since dendrite diameters typically decrease the further they are away from the soma, ER diameters are forced to decrease as well. This would lead to a higher probability for calcium waves to become abortive. Figure 3 demonstrates how such dendritic tapering can be compensated for by an increase in RyR density.
(iii) Conversely, as calcium release from the ER increases both with ER radius and RyR density, there is also a threshold ER radius for any sufficiently large RyR density above which calcium waves are stable. Thus, larger ER compartments in dendrites will enable neurons to more readily induce long-distance calcium signals.
(iv) There are regimes in which abortive calcium waves are initiated (closely below threshold density). Such threshold dependency has previously been introduced in [26]. Our results confirm the existence of such thresholded regimes and are later quantified by deriving empirical laws. While the general behavior will be either a micro-domain calcium event or a calcium wave, there is a critical intermediate region in which abortive waves with variable travel distances can be elicited.

In summary, the interplay between ER radius and RyR density enables dendrites to modulate the stability and reach of calcium signals, which may be critical in synaptic cross-communication and synapse-to-nucleus signaling.

### Threshold for Stable Calcium Waves

Having found the existence of such a threshold, we examined the prerequisites for stable calcium waves more closely. The guiding questions for this investigation were:

1. Do such thresholds also exist for larger dendrites?
2. If so, what is the relationship between dendrite radius and ER radius (and RyR density) at the threshold?

To address these questions, we varied the dendrite radius between 0.1 μm and 1.0 μm and the RyR channel density in the ER membrane between 0.5 $\upmu \mathrm{m}^{-2}$ and 4.0 $\upmu \mathrm{m}^{-2}$. For various combinations of these two parameters, we determined the threshold ER radius, above which a stable calcium wave can be elicited, using a bisection technique: We first located an interval in the ER radius domain for which the lower bound did not allow for a stable calcium wave, while the upper bound did. Then we simulated the calcium dynamics with ER radius taken from the middle of the interval and repeated the procedure with the lower half of the interval, if a stable wave was detected, with the upper half otherwise, until an adequate precision was reached. For this purpose, we accepted as a stable wave any signal, which, upon activation by calcium influx through the left-hand side of the model dendrite, propagated all the way (i.e., 50 μm) along the dendrite and was detected, in a measurement zone located at the right-hand side (see also Fig. 2). With regard to the steep, singularity-like gradients of the wave range/RyR density functions for ranges between 20 μm and 50 μm in Fig. 3, we are confident that this is an appropriate choice. Since even if a setting existed where a wave had a range of 50 μm but became abortive beyond that range, a minor increase in RyR density (or ER radius) would then substantially increase its range.

**Fig. 4:**
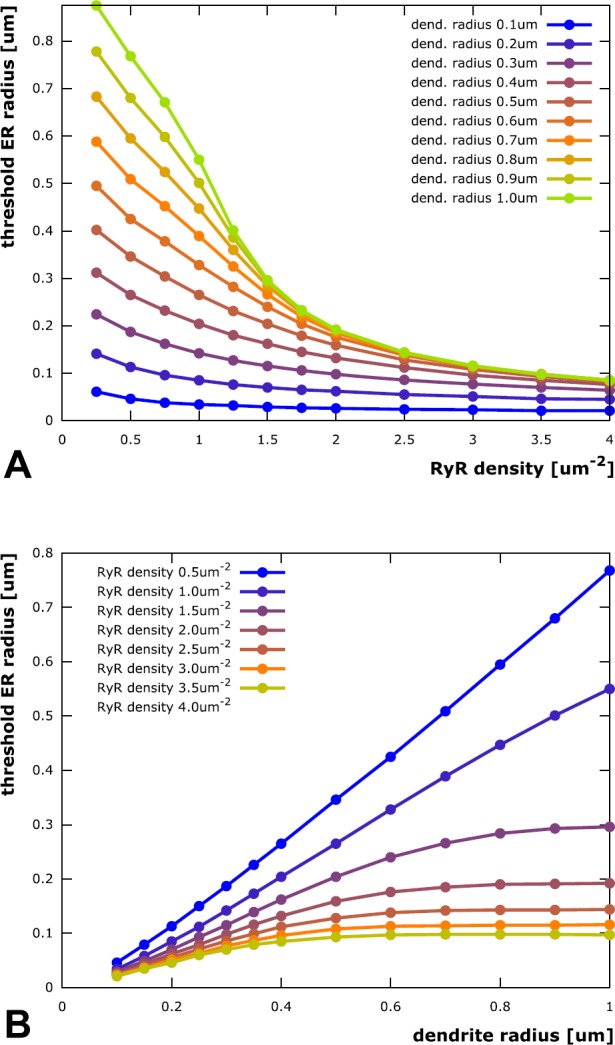
Threshold ER radius for eliciting stable calcium waves. For any tested combination of RyR channel density and dendrite radius, there exists a threshold ER radius above which stable calcium waves can be elicited. Overall, the threshold ER radius increases with increasing dendrite radius (since there is more space that the released calcium can diffuse into, which reduces the effective near-ER-membrane concentration) and decreasing RyR density (indicating that the ability to sustain a stable calcium wave scales with the rate of calcium release from the ER). (**A**) Threshold ER radius as a function of RyR channel density in the ER membrane. The lower four traces, corresponding to the lower dendrite radii, exhibit similar quasi-hyperbolic behavior, scaled by the dendrite radius. They can be fitted by a functional description that assumes instant radial distribution of released calcium in the small radial range from ER to plasma membrane, cf. “Empirical threshold laws”. The upper traces, corresponding to larger dendrites, show two separate regimes: Separated to the left, where the distance between ER and plasma membrane is small, they converge towards a limit trace to the right, where the two membranes are far away from each other. This convergence can be attributed to wave propagation that is faster than radial diffusion to the plasma membrane, cf. again “Empirical threshold laws”. (**B**) Threshold ER radius as a function of dendrite radius (like A, but projected onto perpendicular plane). While the traces are approximately linear in a small dendrite regime, they reach a limit threshold for larger dendrites. No such limit is reached for the lowest RyR density tested. This is due to calcium buffering in the cytosol. Thus, ER size does not need to be increased proportionally with dendrite radius. Neurons could therefore ensure stable calcium waves, even within larger dendrites, where intracellular space is occupied by other organelles
(ii) The traces in Fig. 4B (threshold ER radius plotted against dendrite radius) reach an upper limit for large dendrite radii. In other words, there is a limit to the thickness of the ER required to elicit stable calcium waves and the ER does not need to be bigger than that in, e.g., very thick proximal dendrites. Figure 5 depicts the limit ER threshold values as a function of RyR density. The data is fitted by a model function of the form
  $$\begin{aligned} r_{\mathrm{lim}}(\rho ) = \frac{ac+b\rho }{\rho - c} \end{aligned}$$
  with $a = 0.377\ \upmu \mathrm{m}$, $b = 0.0115 \ \upmu \mathrm{m}$ and $c = 0.637\ \upmu \mathrm{m}^{-2}$.

With a look at the results in Fig. 4, the first question can be answered affirmatively. In fact, for all combinations of dendrite radius and RyR density that we checked, we found an ER radius threshold above which stable calcium waves were produced. This threshold ER radius decreases with increasing RyR density (indicating that the ability to sustain a stable calcium wave scales with the rate of calcium release from the ER) and increases with growing dendrite radius (because there is more space that the released calcium can diffuse into, which reduces the effective near-ER-membrane concentration). It is interesting to point out three more aspects:

(i) The traces in Fig. 4A (threshold ER radius plotted against RyR density) appear to reach a lower limit for large RyR densities, meaning the ER needs to have a minimal size to produce stable calcium waves. This makes sense as it has to contain a minimal amount of calcium that can actually be released during a wave event. This result is in agreement with [36], where an increase in SERCA density led to an increase in luminal calcium. This was demonstrated to have a positive feedback on the wave dynamics.

**Fig. 5:**
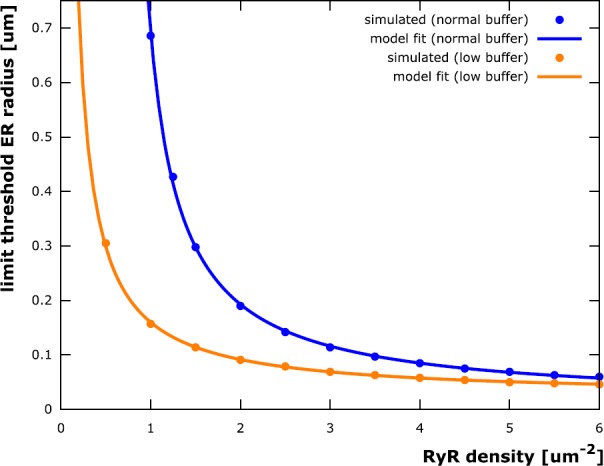
Limit threshold ER radius as function of RyR density. The ER radius *r* above which stable calcium waves can be elicited for arbitrarily large dendrites (for a given RyR channel density *ρ*, cf. Fig. 4) can be fitted by a function of the form $r(\rho ) = \frac{ac+b\rho }{\rho - c}$. The parameter values $a = 0.377 \ \upmu \mathrm{m}$, $b = 0.0115 \ \upmu \mathrm{m}$ and $c = 0.637\ \upmu \mathrm{m}^{-2}$ have been determined using a least squares fitting. The value of *c* indicates that such a limit ER radius exists only for RyR densities larger than approximately 0.64 $\upmu \mathrm{m}^{-2}$. This is due to calcium buffering in the cytosol: Simulations with the cytosolic buffer concentration reduced by 90% result in a fitting curve with $c = 0.007\ \upmu \mathrm{m}^{-2}$. Neurons could therefore modify RyR densities according to intracellular buffering properties to then maintain a minimal ER “footprint” for stable calcium waves
(iii) The trace corresponding to the lowest tested RyR density (0.5 $\upmu \mathrm{m}^{-2}$) in Fig. 4B does *not* reach such an upper limit (the trace corresponding to a RyR density of 1.0 $\upmu \mathrm{m}^{-2}$ does reach a limit, but outside the depicted range). One can conclude that a minimal RyR density is required for stable calcium waves (see also [26]) if the ER is restricted to specific dendritic volumes. Further investigation of this observation showed that this is due to calcium buffering in the cytosol: If the RyR-supported calcium current through the membrane is too small, too much of the released calcium is buffered and there is not enough left to trigger further release through RyR channels. This results is in agreement with [37], where similar conclusions were made for IP_3_R-induced waves. Reducing the buffer concentration by a factor of ten allowed for a limit ER threshold radius for all tested RyR densities (cf. Fig. 5).

#### Empirical Threshold Laws

To complete the investigation of our second question, we tried to describe the relationship between threshold ER radius and dendrite radius/RyR density by deriving appropriate empirical laws for the traces in Fig. 4. The fact that we found a threshold ER radius for wave elicitation in all combinations of dendrite radius and RyR density suggests that there exists a function $r(R,\rho )$ (*r* for ER radius, *R* for dendrite radius, *ρ* for RyR density) describing a threshold ER radius manifold in the three-dimensional parameter space spanned by *r*, *R* and *ρ*.

**Fig. 6:**
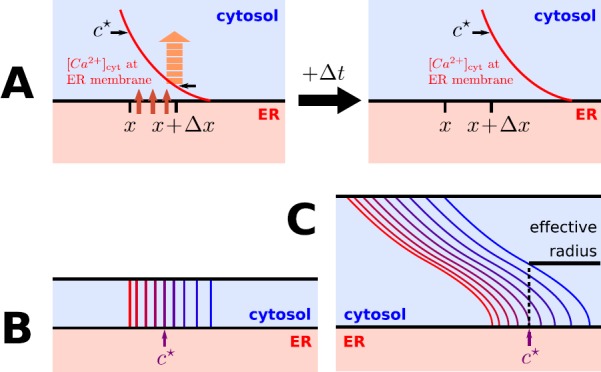
Schematics for deriving empirical threshold laws of wave stability. (**A**) In a stable wave regime the axial position *x*, where a cytosolic threshold calcium concentration $c^{\star }$ is attained near the ER membrane, needs to move by Δ*x* to the right within a given time Δ*t* (defined by the wave velocity). The amount of calcium required to increase the original concentration at $x + \Delta x$ needs to be released from the ER. (**B**) In dendrites where the distance between ER and plasma membrane is small (compared to the diffusive range of calcium for time Δ*t*), the iso-concentration surfaces (indicated by blue-to-red sequence of lines) are nearly radially aligned. This means the released calcium is roughly constant in radial direction. (**C**) In dendrites where the distance between ER and plasma membrane is large (compared to the diffusive range), the iso-concentration manifolds (blue-to-red sequence of lines) are bent backwards in axial direction. Calcium released from the ER only diffuses to an effective radius within the wave propagation time Δ*t* and the wave front does not “sense” the plasma membrane

The spatio-temporal patterns of calcium waves can be roughly separated into three groups by means of the distance between ER and plasma membrane. The first group is defined by morphological configurations in which this distance is small enough for the radial calcium concentration profiles to be approximately constant, the second group contains the cases in which the distance is large enough for it not to influence the wave propagation, and the third group comprises all intermediate cases. In this section, we will derive separate empirical threshold laws for the former two groups. To motivate this distinction, we introduce a little thought experiment (cf. Fig. 6A).

In a stable wave regime, the axial position *x*, where a cytosolic threshold calcium concentration $c^{\star }$ is attained near the ER membrane, needs to shift by Δ*x* to the right within a certain time Δ*t* (governed by the wave velocity). The amount of calcium necessary to increase the original concentration at $x + \Delta x$ to $c^{\star }$ within this time span needs to be released from the ER. It is proportional to the number of releasing RyR channels (see also [26]), which, in turn, is proportional to: (i) the RyR density *ρ* and (ii) the radius *r* of the ER membrane, both assumed constant in time. The single-channel current, however, is time-dependent in that it depends on locally available endoplasmic calcium (see Eq. (8)), which decays rapidly during RyR release. While the amount of released calcium is proportional to *r*, the endoplasmic volume is proportional to $r^{2}$. Assuming rapid radial equilibration within the ER, the decay of a single-channel current is proportional to $r^{-1}$. Integrating the decreasing current from *t* to $t+\Delta t$ shows that the amount of released calcium is also proportional to (iii) the factor $(1-b/r)$ with some constant *b*, in a first-order approximation. Putting (i) to (iii) together, an amount proportional to

$$\begin{aligned} \rho r \biggl( 1-\frac{b}{r} \biggr) \end{aligned}$$

is released into the cytosol, This is where we distinguish between regimes with small and large distances between plasma and ER membrane.

##### Small Distance Between Plasma and ER Membrane

When the distance between ER and plasma membrane is small, then there is only a negligible radial concentration gradient at all times, as diffusion will almost instantaneously produce constant radial concentration profiles (cf. Fig. 6B). With this in mind, the increase in cytosolic calcium concentration, caused by the amount of calcium released from the ER, is inversely proportional to $R^{2}-r^{2}$, assuming that a fixed percentage of the released calcium is bound by the mobile buffer, which leaves proportionality intact. As the required increase to bridge the gap between the concentrations at *x* and $x+\Delta x$ is fix, a constant $\frac{1}{2c}$ can be introduced to arrive at the relation

$$\begin{aligned} \frac{\rho (r-b)}{R^{2}-r^{2}} &= \frac{1}{2c} \\ \quad \Leftrightarrow\quad r &= -c\rho + { \bigl( (c\rho )^{2} + 2bc \rho + R^{2} \bigr) } ^{0.5} \end{aligned}$$

with the parameters *b* and *c*, usable in a least squares fitting, which very closely fits the 0.1 μm dendrite radius trace in Fig. 4A. The one for the 0.2 μm dendrite radius trace is also acceptable, but using *R* as an additional parameter in the fitting expression produces substantially better fitting results for the 0.3 μm and 0.4 μm dendrite radius traces, indicating that the model assumption of radially constant calcium concentrations is already largely violated in theses cases.

##### Large Distance Between Plasma and ER Membrane

When the distance between ER and plasma membrane is large, the wave front moves faster in the axial direction than its radial component reaches the plasma membrane, and the wave does not “sense” the presence of the plasma membrane (cf. Fig. 6C). The fact that the upper traces in Fig. 4A converge towards a limit trace and that the lower traces in Fig. 4B attain a limit is due to this circumstance. In this situation, radial calcium diffusion will be limited to a distance *a* from the ER membrane until the threshold concentration $c^{\star }$ is reached at axial position $x+\Delta x$. It is reasonable to assume that the calcium concentration near the ER membrane after release through RyR channels is inversely proportional to the volume it diffuses into, i.e., $(r+a)^{2} - r^{2}$. Like above, with a fixed percentage of the released calcium being bound by the buffer, we can introduce a constant $\frac{c}{4a}$ (which depends on the buffer concentration) to arrive at the equation

$$\begin{aligned} \frac{\rho (r-b)}{(r+2a)^{2}-r^{2}} &= \frac{c}{4a} \\ \quad \Leftrightarrow \quad r &= \frac{ac+b\rho }{\rho -c}. \end{aligned}$$

The parameters *a*, *b*, and *c* can be determined to fit the limit threshold ER radii attained for large dendrite radii (Fig. 5, blue trace), also for buffer concentrations reduced by a factor of ten, in which case the parameter *c* is significantly smaller (Fig. 5, orange trace). Note that the buffer concentration puts a constraint on the RyR density: If the density falls below a threshold (the constant *c*), then there can be no calcium wave, regardless of how big the ER is.

##### Intermediate Distances

We were not able to find descriptive model functions to fit the threshold values for intermediate distances. In such regimes, the significant radial concentration gradient at the wave front, which varies strongly with the distance between the two membranes, makes it difficult to find expressions similar to the two presented ones.

### Wave Velocity

**Fig. 7:**
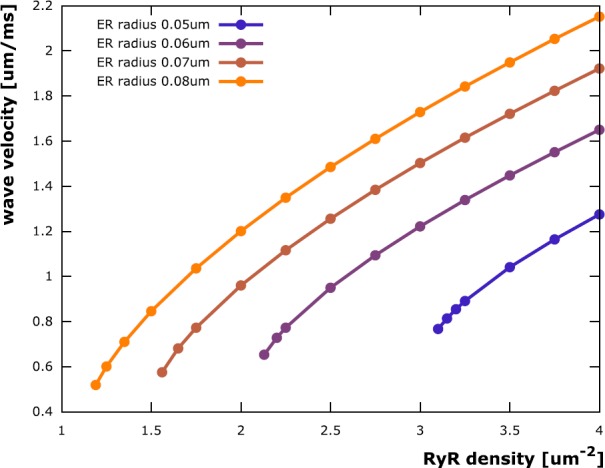
Stable calcium wave velocity. The velocity of stable calcium waves depends on the RyR channel density in the ER membrane and on the ER radius. The traces for wave velocity as a function of RyR density rise quickly in a small range above the threshold for stable waves and then exhibit a quasi-linear behavior with a slope seemingly independent of the ER radius. In dendrites where multiple synaptic sites are activated in a spatio-temporal manner, this linear dependence could result in linear additivity of multiple calcium wave velocities and thus may be useful in describing multi-wave interactions in more complex dendritic branching patterns

During the described simulations, we observed that the velocity of calcium waves varies with ER radius and RyR density. This led us to finally examine the velocity of stable waves. Following the initial activation, all stable waves reached a constant velocity shortly after their formation (cf. Fig. 2C)). These constant velocities were measured using the rightmost axial coordinate where the RyR open probability exceeded 0.1 as the wave front position in every time step of the simulation. Results (shown for a dendrite radius of 0.2 μm in Fig. 7) indicate that calcium wave velocity increases both with increasing ER radius and RyR density. This can be expected given that the driving force for the movement of the wave is diffusion, which becomes faster when axial concentration gradients become bigger, which, in turn, is the case when the efflux density from the ER increases. The traces for wave velocity as a function of RyR density rise more rapidly in a small range above the threshold for stable waves and then exhibit a quasi-linear behavior with a slope seemingly independent of the ER radius. Velocity traces for a larger dendrite of radius 1.0 μm show the same basic behavior (data not shown), though with a less pronounced dependency on the ER radius, which is in line with our findings for the ER radius threshold in the previous section.

## Discussion

Our computational study finds that calcium waves can be triggered in an idealized morphological setting. If certain geometric and physiological conditions are met, wave propagation is a stable process, similar to the all-or-nothing properties of electrical action potentials. A minimal ryanodine receptor density is required to guarantee sufficient calcium release from the ER and this density scales inversely with ER surface. We also observed abortive regimes in small parameter regions below thresholds for ER size and ryanodine receptor densities. When considering realistic morphologies, spatial variations in dendritic and ER morphology as well as changes in RyR density are likely to occur, thus, abortive regimes may be important when it comes to synaptic cross-communication with respect to calcium signals.

We used spatially constant RyR densities and membrane radii throughout the study, since we were interested in obtaining functions expressing their relationships with respect to the potential for the elicitation of stable calcium waves. Of course, in real dendrites neither dendrite radius nor RyR density are spatially constant [64], nor can the ER be expected to be a perfectly shaped cylinder that is centrally positioned within the dendrite. Future research, which is outside the scope of this study, could integrate heterogeneous membrane composition and detailed three-dimensional morphologies.

In the context of spike-timing-dependent plasticity, calcium wave velocity may be a controlling parameter. Our study shows that calcium wave velocities vary depending on ER size and RyR density. This merits a closer investigation of the timing of calcium signals in connection to, e.g., back-propagating action potentials.

A more precise understanding of the intracellular architecture may be critical in wave timing and stability. For example, mitochondria are an important sink for excess cytosolic calcium. They can quickly absorb considerable amounts of calcium through mitochondrial calcium uniporter channels [65] and extend through dendrites as filamentous networks [41, 42]. It would therefore be interesting for future work to extend this study to a fully three-dimensional, non-symmetric case, to investigate the interplay between mitochondria and ER in the context of dendritic calcium waves.

In addition to the stability and velocity of dendritic calcium waves, changes in the cellular and intracellular architecture may promote direction selectivity of calcium signals. Since the radius of dendrites is variable, typically large in proximity of the soma and smaller in more distal regions, we wondered whether there could be some kind of direction selectivity in the propagation of calcium waves. Due to the increasing amount of releasable calcium in the growing ER, we suspected that stable waves might be better supported when propagating from thin dendrites to larger ones with larger ER than in the direction of decreasing radii. We tested this hypothesis with three simulations (data not shown), the first on a medium-size dendrite with constant radius, the second on the same dendrite with increasing radius, and the last one on the same dendrite with decreasing radius. In all cases, the ER radius at each location was chosen to be just below the threshold for stable calcium waves at the chosen (constant) RyR density, so that one would expect the waves to terminate at some point. This indeed happened in all cases, however, the distance the waves were able to travel differed: shortest in the shrinking dendrite and longest in the expanding dendrite. The results confirmed our hypothesis. Yet, the effect was relatively small (approx. 11 μm difference in travel distance between small-to-large and large-to-small scenarios) and required very precise calibration of the ER radius (too small and all waves would have terminated even earlier, too large and all waves would have been stable). We therefore believe that this effect is unlikely to be relevant under realistic conditions.

Our study further highlights the question of model dimensionality. While in many cases, in which electrical properties of neurons are studied, one-dimensional multi-compartment models are employed, this may not always be possible for intracellular biochemical modeling. The fact that we could not find a simple descriptive model for regimes with neither very small nor very large distance between ER and plasma membrane reflects the non-trivial dynamics of the calcium signal with respect to space. Even in this relatively simple model setup with rotational symmetry in the geometry, it is not generally possible to reduce dimensionality (by ignoring the radial concentration profile in the cytosol) to a model that is essentially 1D. A fortiori, such a model would not be able to generate the correct stability thresholds and wave velocities in medium-size and large dendrites. For very thin dendrites, however, a simplification of 3D calcium models to 1D is conceivable provided the symmetry prerequisites of this study are met.

## Electronic Supplementary Material

Below are the links to the electronic supplementary material.

Video of wave propagation in thin dendrite: Typical wave in a dendrite of radius 0.2 μm, with an ER radius of 50 nm and a ryanodine receptor density in the ER membrane of 3.5 $\upmu \mathrm{m}^{-2}$. One second in the video corresponds to 10 ms of simulated time. Calcium concentrations are color-coded blue ($c _{c}\leq 50\ \mathrm{n}\mathrm{M}$, $c_{e}\leq 25 \ \upmu \mathrm{M} $) to red ($c_{c}\geq 1 \ \upmu \mathrm{M} $, $c_{e}\geq 250\ \upmu \mathrm{M} $). Radial coordinate scaled by a factor of 40 to enhance visibility (MKV 74 kB)

Video of wave propagation in thick dendrite: Typical wave in a dendrite of radius 1.0 μm, with an ER radius of 0.2 μm and a ryanodine receptor density in the ER membrane of 2.5 $\upmu \mathrm{m}^{-2}$. One second in the video corresponds to 10 ms of simulated time. Calcium concentrations are color-coded blue ($c_{c}\leq 50 \ \mathrm{n}\mathrm{M}$, $c_{e}\leq 25 \ \upmu \mathrm{M} $) to red ($c_{c}\geq 1\ \upmu \mathrm{M} $, $c_{e}\geq 250\ \upmu \mathrm{M}$). Radial coordinate scaled by a factor of 8 to enhance visibility (MKV 88 kB)

## Abbreviations

CalB: calbindin-D_28k_
CICR: calcium-induced calcium release
ER: endoplasmic reticulum
IP_3_: inositol 1,4,5-trisphosphate
IP_3_R: IP_3_ receptor
LIMEX: linearly-implicit extrapolation
NCX: Na^+^/Ca^2+^ exchanger
PMCA: plasma membrane Ca^2+^-ATPase
RyR: ryanodine receptor
SERCA: sarco-/endoplasmic reticulum Ca^2+^-ATPase
